# Immunotherapy With Bacillus Calmette-Guerin (BCG) in a 16-Year-Old With Urothelial Bladder Carcinoma

**DOI:** 10.7759/cureus.24994

**Published:** 2022-05-14

**Authors:** Jose M Martinez-Thomas, Luis F Galicia-Belaunzaran, Claudio E Merayo-Chalico, Joseph Palatchi, Juan Carlos Angulo-Lozano

**Affiliations:** 1 Department of Urology, Hospital Angeles Universidad, Mexico City, MEX; 2 School of Medicine, Universidad Anahuac Mexico, Mexico City, MEX; 3 Department of Urology, Hospital General de Mexico, Mexico City, MEX

**Keywords:** transurethral resection of bladder tumor (turbt), urothelial bladder cancer, pediatric oncology, pediatric urothelial cancer, urothelial malignancy

## Abstract

Urothelial bladder cancer (UBC) is an exceptionally rare condition in adolescents between 15 and 19 years of age. Typically, adolescents and pediatric patients with UBC are more likely to have a favorable histological report. The aim of the paper is to report our experience in the management of a 16-year-old patient with UBC with no risk factors that came to the office because of a history of painless gross hematuria.

## Introduction

Urothelial bladder cancer (UBC) is an infrequent condition in adolescents between 15 and 19 years of age, accounting for 0.04% of the population with bladder cancer [1]. UBC typically shows a peak incidence in the sixth or seventh decade of life. Risk factors in the pediatric population include early tobacco exposure, environmental toxins, syndromes that predispose to malignancies, anatomic bladder defects, and parasitic infections (e.g., *Schistosoma haematobium*) [2]. Typically, adolescents and pediatric patients with UBC are more likely to have a favorable histological report and better prognosis than adults [3]. The aim of the paper is to report our experience in the management of a 16-year-old patient with UBC with no risk factors that came to the office because of a history of painless gross hematuria.

## Case presentation

**Figure 1:**
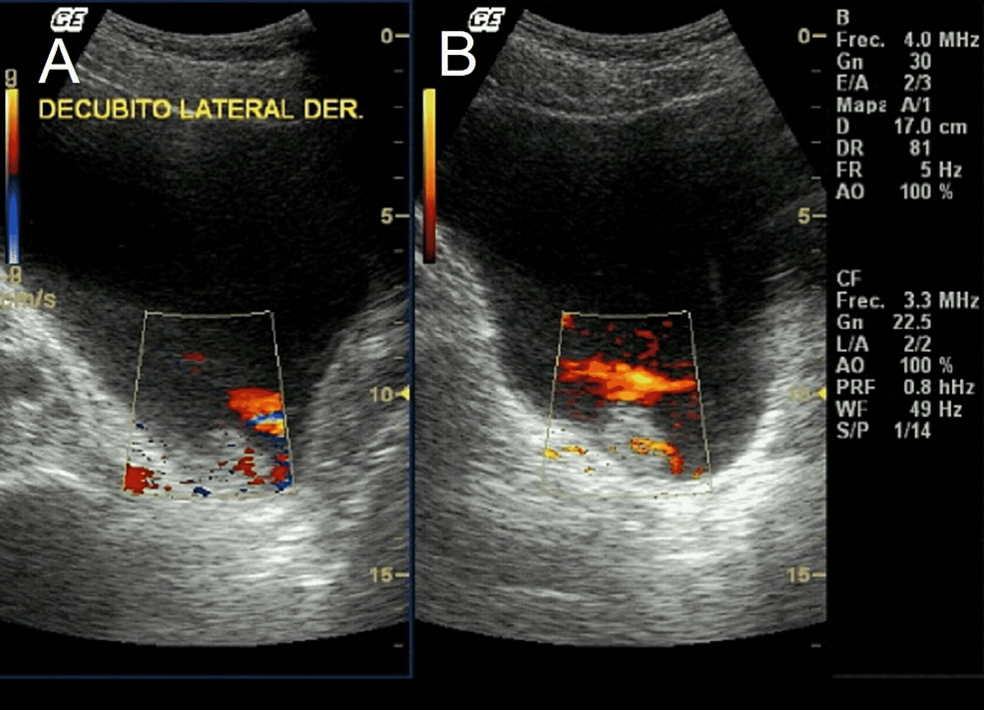
Doppler USG of the bladder showing a mass at the right bladder wall with the presence of ureteral jets on (A) sagittal view and (B) transverse view. USG: ultrasonography

A 16-year-old male high school student came to the office with his parents referred by a primary care physician because of a history of one month of painless gross hematuria. During initial interrogation, family history was negative for cancer and chronic diseases. He denied recent trauma, recent infections, abdominal pain, weight loss, dysuria, urgency, fever, fatigue, or tenesmus. On private interrogation, he denied sexual activity, smoking, alcohol intake, and use of drugs. The physical examination was unremarkable. The abdomen was soft and non-tender, with no masses or signs of visceromegaly. The genitals were normal for age and sex. His height was 1.71 meters, weight 69 kg, BMI was 23.63 kg/m^2^, blood pressure (BP) was 109/71 mmHg, heart rate (HR) was 71 beats/minute, and respiratory rate (RR) was 18 breaths/minute. Suprapubic ultrasonography (USG) revealed a heterogeneous intravesical mass measuring 1.4 cm x 1.1 cm (Figures 1A, 1B).

Abdominal CT (computed tomography) scan showed focal thickening of the right wall of the bladder with dimensions of 1.3 cm x 1.0 cm with enhancement after the administration of intravenous contrast, organs of the reproductive system without alterations. Retroperitoneal and mesenteric lymph nodes had normal characteristics. Initial bloodwork is shown in Table 1 and urinalysis is shown in Table 2.

**Table 1:** Blood test results on initial evaluation.

Parameters	Results
Erythrocytes	4.9 million/mm^3^
Hemoglobin	14.2 mg/dL
Hematocrit	42%
Mean corpuscular volume	84 fL
Leukocytes	6.8 x 10^3^/mm^3^
Platelets	348 x 10^3^/mm^3^
Glucose	97 mg/dL
Urea	11.2 mg/dL
Creatinine	0.8 mg/dL
LDH	193 U/L
PT	10.3 sec
PPT	36.8 sec

**Table 2:** Urinalysis results of the patient.

Parameters	Result
Glucose	Negative
Protein	+1
Ketones	Negative
Specific gravity	1.021
pH	7.8
Nitrite	Negative
Color	Red
Leukocyte esterase	Negative
Bacteria	Negative
Blood	40-50 HPF
Leukocytes	5 HPF
Casts	None

**Figure 2:**
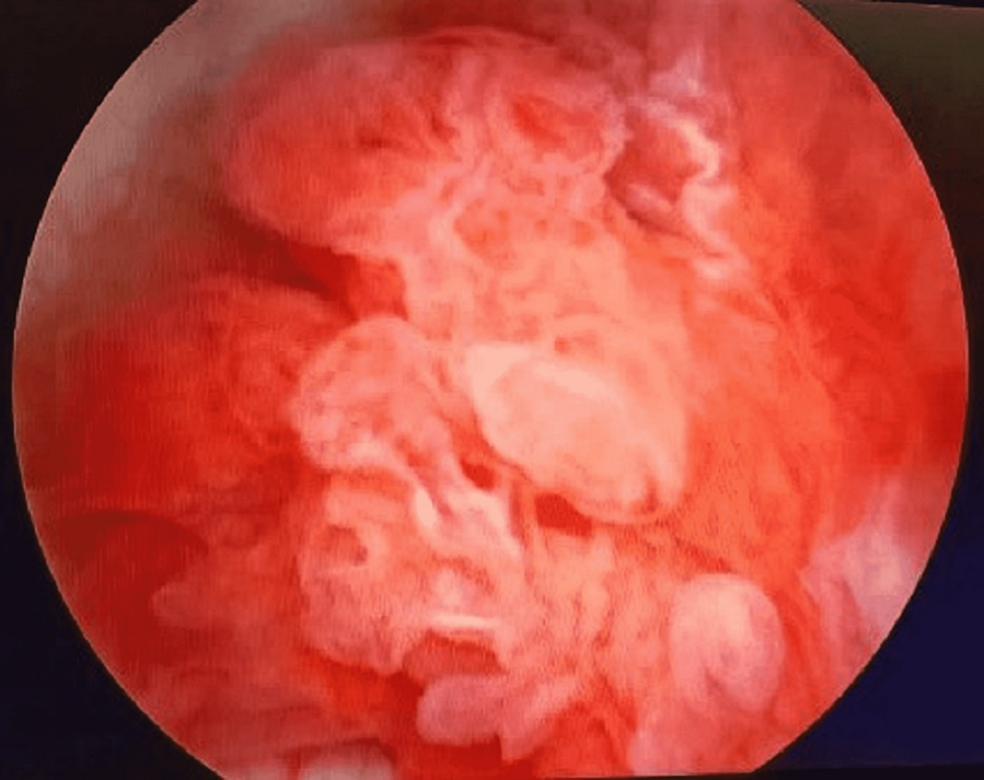
Cystoscopic visualization of the tumor.

**Figure 3:**
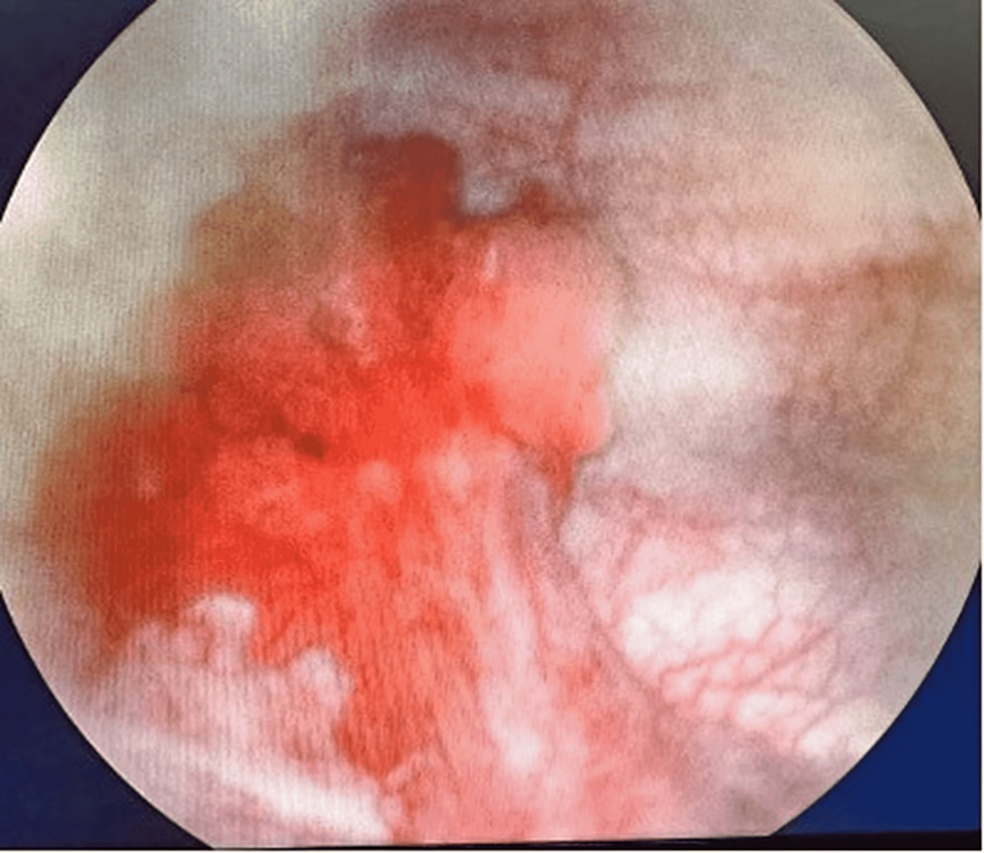
Lateral cystoscopic view of the tumor.

A cystoscopy was conducted under general anesthesia. A tree-shaped tumor was observed on the floor of the bladder, with vascularity and free mobility (Figures 2, 3). Obstruction of the urinary meatus was not observed. Tumor resection was performed, obtaining two samples: the first specimen was labeled as bladder tumor (deep cut), which contained a fragment of nodular tissue that measures 0.7 x 0.5 x 0.5 cm with an irregular surface and brown color. The second specimen was labeled as bladder tumor superficial cut, which had a fragment of tissue that measured 1.2 x 1.2 x 1 cm with an irregular shape and hairy-like surface. Histological findings revealed a transurethral resection product with a neoplasm formed by papillae, some with cellular fusion, lined by mostly monotonous cells with moderate cytoplasm, round nuclei with focal loss of polarity, and increased nucleus/cytoplasm ratio. The lesion had an endophytic growth without infiltration into the muscular layer.

The immunohistochemical report was positive for tissue-specific transcription factor, tumor suppressor protein Ki67. Tumor cell metastasis was not found in the bladder or dissected lymph nodes. Pathology reported a low-grade non-muscle invasive urothelial bladder carcinoma. Intravesical immunotherapy with bacillus Calmette-Guerin (BCG) started six weeks after transurethral resection of bladder tumor (TURBT) and was administered weekly for six weeks. The maintenance dose was administered once at three, six, and 12 months after TURBT. The patient did not experience dysuria, hematuria, or fever during the 12 months. USG at six and 12 months was normal with no evidence of recurrence.

## Discussion

UBC in adolescents is a rare malignancy and is not usually associated with risk factors. The presented case of this 16-year-old boy with no previous history of exposure to tobacco or chemicals and no family history of cancer, with only a one-month history of gross hematuria, makes this case a particular presentation of UBC within a group of age with an extremely low incidence of the disease [1]. Some genetic and congenital syndromes have been linked to UBC like Costello syndrome, Turner syndrome, and Hinman syndrome [4-6].

The main symptom of pediatric patients with UBC is painless macroscopic hematuria. There is a wide range of other unusual presenting symptoms that were reported in literature like urinary frequency, recurrent cystitis, abdominal pain, recurrent urinary tract infections, and vomiting [7]. We performed ultrasonography study as the first diagnostic step to determine the origin of the hematuria. The use of CT scan in young patients is still controversial because of the costs and the risk of radiation exposure [8].

Most of the reported cases are low-grade carcinomas and the available data suggest an excellent prognosis. There is no defined protocol for follow-up for pediatrics with UBC. Ultrasound is the most widely used diagnostic tool for low-grade tumors since it is easy to perform and is not invasive [7-9]. Another tool that can be used is cystoscopy, although it is more invasive.

The guidelines for the treatment of UBC in pediatric population are lacking, there is not a consensus on the literature for the treatment of UBC in pediatric patients, some authors follow the same protocols used in the adult population and other authors tend to use a less aggressive approach as the local recurrence and progression of the tumor tend to be lower compared to the adult population [2,10].

Transurethral resection of bladder tumor (TURBT) is the main treatment for pediatric UBC and has been performed for initial management in more than 95% of the cases. Post-therapy TURBT has not been studied and there are only a few cases to evaluate the efficacy of BCG therapy in the pediatric population. There is also no information in the literature about the monitoring after initial treatment, but some expert recommendations suggest the use of intravesical therapy as it has been shown to be superior compared to intravesical chemotherapy in a case series [11]. Although some authors suggest a more minimalistic approach after TURBT and active follow-up with USG, CT scan, and cystoscopy in three to six months intervals because of the relatively low rates of recurrence (3.8%) [12,13].

## Conclusions

There is still not a consensus about the treatment of non-muscular invasive urothelial bladder carcinoma in the pediatric population, as it is a very rare condition in this group of patients. Expert recommendations differ as some argue that adjuvant immunotherapy is not needed because the majority have a low-grade and low possibility of recurrence. We recommend the use of immunotherapy to minimize the risk of recurrence even if the pathology report is favorable. The use of BCG in this 16-year-old patient was successful and did not present adverse effects.
